# Fluoxetine Pretreatment Promotes Neuronal Survival and Maturation after Auditory Fear Conditioning in the Rat Amygdala

**DOI:** 10.1371/journal.pone.0089147

**Published:** 2014-02-13

**Authors:** Lizhu Jiang, Chen Liu, Jianbin Tong, Rongrong Mao, Dan Chen, Hui Wang, Jufang Huang, Lingjiang Li

**Affiliations:** 1 Mental Health Institute, The Second Xiangya Hospital, and Key Lab of Psychiatry and Mental Health of Hunan Province, Central South University, Changsha, China; 2 Department of Anatomy and Neurobiology, School of Basic Medical Sciences, Central South University, Changsha, China; 3 The Third Xiangya Hospital, Central South University, Changsha, China; 4 Key Lab of Animal Models and Human Disease Mechanisms, Kunming Institute of Zoology, the Chinese Academy of Sciences, Kunming, China; 5 Department of neuropsychopathy, clinical medical school, Dali University, Dali, China; Radboud University, Netherlands

## Abstract

The amygdala is a critical brain region for auditory fear conditioning, which is a stressful condition for experimental rats. Adult neurogenesis in the dentate gyrus (DG) of the hippocampus, known to be sensitive to behavioral stress and treatment of the antidepressant fluoxetine (FLX), is involved in the formation of hippocampus-dependent memories. Here, we investigated whether neurogenesis also occurs in the amygdala and contributes to auditory fear memory. In rats showing persistent auditory fear memory following fear conditioning, we found that the survival of new-born cells and the number of new-born cells that differentiated into mature neurons labeled by BrdU and NeuN decreased in the amygdala, but the number of cells that developed into astrocytes labeled by BrdU and GFAP increased. Chronic pretreatment with FLX partially rescued the reduction in neurogenesis in the amygdala and slightly suppressed the maintenance of the long-lasting auditory fear memory 30 days after the fear conditioning. The present results suggest that adult neurogenesis in the amygdala is sensitive to antidepressant treatment and may weaken long-lasting auditory fear memory.

## Introduction

Fear is an emotion conserved in the animal kingdom as an instinctual response to potential danger, and is critical for adaptive functions of the brain. However, persistent fear can lead to maladaption and even stress-related disorders such as phobias and post-traumatic stress disorder (PTSD) [1], [2]. Intensive studied over many years has revealed a fundamental understanding of the underlying mechanisms of increased stress sensitivity, overconsolidation and reconsolidation of fear memories and impairment of fear extinction [3], [4]. It is well known that persistent fear associated long-term memory engages gene expression, new protein synthesis and synaptic plasticity [5], [6]. Further, adult neurogenesis could enable the synaptic remodeling of mature neuronal circuits, contributing to structural and functional plasticity in the brain. However, it is unclear whether adult neurogenesis in the amygdala is involved in the maintenance of long-term auditory fear memory.

Adult neurogenesis has been clearly demonstrated in the subgranular zone (SGZ) of hippocampal dentate gyrus (DG) and the subventricular zone (SVZ) of the lateral ventricles. Environmental factors including environmental enrichment, physical exercise, aging and stress can positively or negatively modulate adult hippocampal neurogenesis [7]. Previous studies have shown that adult neurogenesis in the DG is involved in the contextual fear conditioning [8], [9], spatial pattern separation [10] and spatial memory [11], [12]. Impaired adult neurogenesis in the hippocampus prolongs the hippocampus-dependent time period of associative fear memory [9] and inhibits the formation of hippocampus-dependent contextual fear memory [8].

The amygdala contributes to the acquisition and storage of fear memory [4], [13], [14]. Interestingly, adult neurogenesis has also been detected in the amygdala of monkeys, rats and mice [15]–[17]. Similar to the hippocampus, adult neurogenesis in the amygdala is increased by environmental enrichment [17], but impaired by behavioral stress such as social isolation [18]. However, it is still unclear whether adult neurogenesis in the amygdala contributes to auditory fear memory in a manner analogous to adult neurogenesis in the hippocampus contributing to hippocampus-dependent memory. The antidepressant fluoxetine (FLX), a selective serotonin reuptake inhibitor (SSRI), can increase adult neurogenesis in the hippocampus [19]–[21]. Combined with extinction training, chronic administration of FLX before or after fear conditioning can erase fear memory in rats [22]. In the present study, we first observed that auditory fear conditioning decreased neuronal survival and altered neuronal differentiation in the amygdala and hippocampus. A lower proportion of BrdU-positive neurons expressed a marker for mature neurons, NeuN, after auditory fear conditioning. In contrast, the proportion of astrocytes generated from new-born cells was increased after auditory fear conditioning. Chronic treatment with FLX ameliorated the changes in adult neurogenesis induced by auditory fear conditioning and led to an improvement in the long-lasting auditory fear memory.

## Materials and Methods

### Animals

Adult male Sprague–Dawley rats (9 weeks old, inbred strain, Animal House Center, Central South University, Changsha) weighting 200–250 g were used. Rats were group-housed with *ad libitum* access to water and food (12 h light/dark cycle) under controlled (22±2°C) conditions. All experiments were carried out between 09∶00 and 17∶00. The animal care and the experimental protocol were approved by the Animal Ethics Committee of Central South University.

### Drug Administration

Animals were randomly divided into four groups: control, fear, saline fear (Sal+F), and FLX fear (FLX+ F) groups (n = 48 for each group). Rats in the Sal+F group received a saline gavage for 2 w before auditory fear conditioning, and rats in FLX+ F group received a FLX gavage (10 mg · kg^−1^ · d^−1^; solution in normal saline, Watson pharmaceuticals (changzhou) co., LTD) for 2 w before auditory fear conditioning. Three days before anditory fear conditioning, some of the rats in each group (n = 19 to 20) were intraperitoneally injected with 200 mg · kg^−1^ bromodeoxyuidine (BrdU; Sigma) twice to label newborn cells. The administration interval was 12 hours.

### Auditory Fear Conditioning

Three days after BrdU administration, rats in the fear, Sal+ F and FLX+ F groups were placed into a conditioning chamber (30 cm × 24 cm × 21 cm) that was enclosed in a ventilated and sound-attenuated box (63 cm× 43 cm× 63 cm) for 2 min and recieved 20 trials of a neutral tone (80 dB, 20 s, 2 kHz) co-terminating with an electrical foot shock(0.75 mA, 5 s), the inter-trial interval was 90 s. One min after the last shock, animals were returned to their home cage. Rats in the control group were processed with the same protocol except that the footshock was omitted. For maximal conditioning, the twenty tone-shock pairings were repeated on the next day. During the retrieval test, a Plexiglas chamber distinct from the conditioning chamber was used (chamber B). Rats were placed into chamber B and exposed to three tone-alone presentation (80 dB, 20 s, 2kHz; 90-s intertrial interval) at 1 d, 7 d, 14 d, 21 d, 30 d or 60 d after training. Freezing behavior was defined as immobility except for respiratory movements and was quantified by trained observers that were blind to the experimental groups. The BrdU-labled rats were sacrificed immediately after the retrieval test to examine neurogenesis.

### Immunohistochemistry and Confocal Imaging

Rats injected with BrdU were anesthetized (pentobarbital 40 mg/kg, IP) and fixed by perfusing with ice-cold 4% paraformaldehyde in 0.1 M phosphate buffer (pH 7.4). The brains were removed and postfixed overnight in 4% paraformaldehyde at 4°C. After treatment with 30% sucrose, brains were cut into sections (40-µm thickness) through the entire amygydala and hippocampus (Paxinos and Watson, 1998) in the coronal plane. For stereological BrdU counts, 1 of every 12 sections throughout the entire amygdala and hippocampus were mounted onto slides, heated in citric acid (0.1 M, pH 6.0) for 30 min at 95°C for antigen retrieval, and denatured in 2 N HCl for 30 min at 37°C. Sections were incubated for 15 min in 3% hydrogen peroxide to eliminate endogenous peroxidases, then rinsed and incubated with blocking buffer (5% bovine serum albumin, 0.3% Triton X-100) for 1 h. Sections were then incubated with a mouse anti-BrdU antibody (1∶1000; Sigma) overnight at 4°C, rinsed and transferred to biotinylated goat anti-mouse IgG (1∶200; Vector Laboratories) at room temperature for 2 h. BrdU was visualized using an avidin-biotin-horseradish peroxidase kit (Vector Laboratories) and reacted with diaminobenzidine (Sigma).

For double labeling, immunofluorescence staining was used. Briefly, denatured sections were incubated overnight with rat anti-BrdU monoclonal IgG (1∶500, Accurate Chemical) and a mouse anti-NeuN monoclonal IgG (1∶400; Millipore), or a goat anti-GFAP polyclonal IgG (1∶1000; Santa Cruz) in PBS. The secondary antibodies were Cy3-conjugated donkey anti-rat IgG (1∶500; Jackson ImmunoResearch), and Alexa Fluor 488 goat anti-mouse IgG (1∶500, Jackson ImmunoResearch) or Alexa Fluor 488 Rabbit anti-goat IgG (1∶500, Jackson ImmunoResearch). Sections incubed in the secondary antibodies for 1 h and coverslipped with fluorescent mounting medium (Invitrogen). Fluorescence images were captured on a confocl microscope (Nikon, DIGITAL ECLIPSE C1 plus, Japan).

### Cell Counting

The number of BrdU-positive cells was estimated using a modified stereology protocol [23]. Every twelfth section throughout the entire amygdala (including the basolateral amygdaloid nucleus, basomedial amygdaloid nucleus, medial amygdaloid nucleus and cortical amygdaloid nucleus) and hippocampus were processed for BrdU immunohisochemistry. All BrdU-positive cells were counted by an observer using a 40 × objective, and the observer was blinded to the experimental design. The number of labeled and counted cells was multiplied by 12 to obtain an estimate of the total (bilateral) number of BrdU positive cells per animal.

#### Co-localization of BrdU/NeuN and BrdU/GFAP markers

For estimating the phenotype of newborn cells, slices were analyzed on a confocal microscope (Nikon, DIGITAL ECLIPSE C1 plus, Japan), Single-layer images were scanned, and Z-stacks of confocal images (at 1 µm intervals) were projected to reconstruct astrocyte structure [24]. Maker expression was examined for 40–60 BrdU-positive cells per animal [25].

### Statistical Analysis

Data are presented as group means ± SEM. Group difference were assessed with Student’s t-test or analysis of variance (ANOVA). The criterion for significance was set at *P*<0.05.

## Results

### Auditory Fear Conditioning Decreased the Survival of the Adult-born Cells in the Amygdala

**Figure 1:**
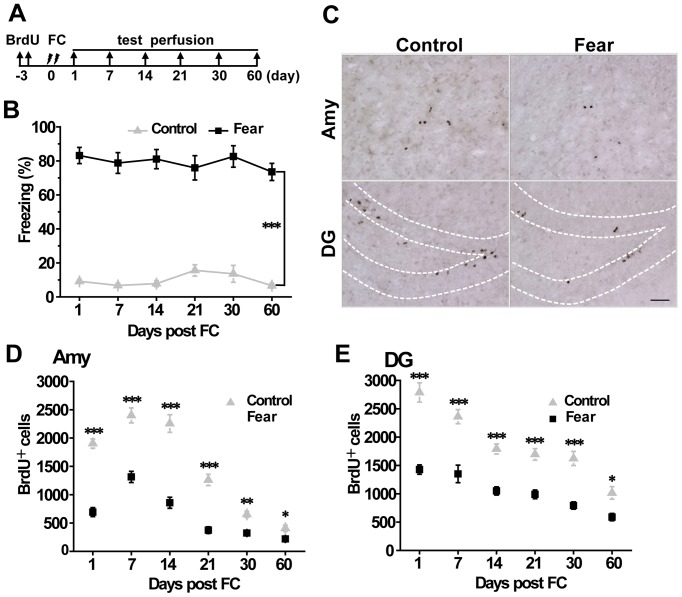
Auditory fear conditioning induced persistent fear memory and decreased the survival of adult-born cells in both the amygdala and hippocampus. (A) Diagram of experimental procedures. (B) Auditory fear conditioning induced long-term persistent fear memory. Compared to the control animals, the freezing level in fear rats was significantly increased at 1 d, 7 d, 14 d, 21 d, 30 d and 60 d after fear conditioning (n = 8). (C) Representative photomicrographs of BrdU immunostaining in the dentate gyrus of the hippocampal subfield and the amygdala in controls and fear rats one day after auditory fear conditioning. Auditory fear conditioning significantly suppressed the survival of new born cell in both the amygdala (n = 4) (D) and the hippocampus (n = 4) (E). FC = fear conditioning; DG = dentate gyrus; Amy = amygdala. * *P*<0.05, ** *P*<0.01, *** *P*<0.001. Scale bar = 50 µm.

We tested the hypothesis that a protocol that produced strong auditory fear memory could alter adult neurogenesis in the amygdala (Figure 1A). Rats in the fear group received 20 trials of a neutral tone (80 dB, 20 s, 2 kHz) co-terminating with an electrical footshock (0.75 mA, 5 s), while rats in the control group were subjected to the same protocol without the footshock. For the maximal conditioning, the twenty tone-shock pairings were repeated on the next day. Auditory fear memory was examined at 1 d, 7 d, 14 d, 21 d, 30 d or 60 d after the conditioning. Compared with the marginal freezing of controls, auditory fear memory in the fear-conditioned rats were significantly heightened at 1 d (fear vs. control: 83.16±4.73% vs. 9.17±1.18%, *P*<0.001), 7 d (fear vs. control: 78.75±6.11% vs. 6.76±1.25%, *P*<0.001), 14 d (fear vs. control: 81.4±5.64% vs. 7.83±1.79%, *P*<0.001), 21 d (fear vs. control: 75.92±7.17% vs. 15.6±3.34%, *P*<0.001), 30 d (fear vs. control: 82.60±6.32% vs. 13.58±4.98%, *P*<0.001) and 60 d (fear vs. control: 73.53±5.03% vs. 6.63±1.45%, *P*<0.001) after the fear conditioning (Figure 1B). These dada demenstrate that the exprimental protocol of auditory fear memory was appropriate for studying adult neurogenesis in the amygdala.

We next examined whether anditory fear conditioning could affect adult neurogenesis in the amygdala and the hippocamopus by immunohistochemistry (Figure 1C). Remarkably, we found that auditory fear conditioning significantly decreased the number of BrdU-positive cells in the amygdala at 1 d (control vs. fear: 1904±81.68 vs. 693±79.22; *P*<0.001), 7 d (control vs. fear: 2400±231.96 vs.1314±98.71; *P*<0.001), 14 d (control vs. fear: 2256±155.53 vs. 858±97.24; *P*<0.001), 21 d (control vs. fear: 1260±99.43 vs. 375±58.66; *P*<0.001), 30 d (control vs. fear: 654±54.88 vs. 324±34.29; *P*<0.01) and 60 d (control vs. fear: 408±55.53 vs. 219±20.42; *P*<0.05) after auditory fear conditioning (Figure 1D). Similar results were also observed in the hippocampus following auditory fear conditioning (Figure 1E). Behavioral stress may be critical because the number of BrdU-positive cells was significantly decreased.

### Auditory Fear Conditioning Altered the Differentiation of Adult-born Cells

**Figure 2:**
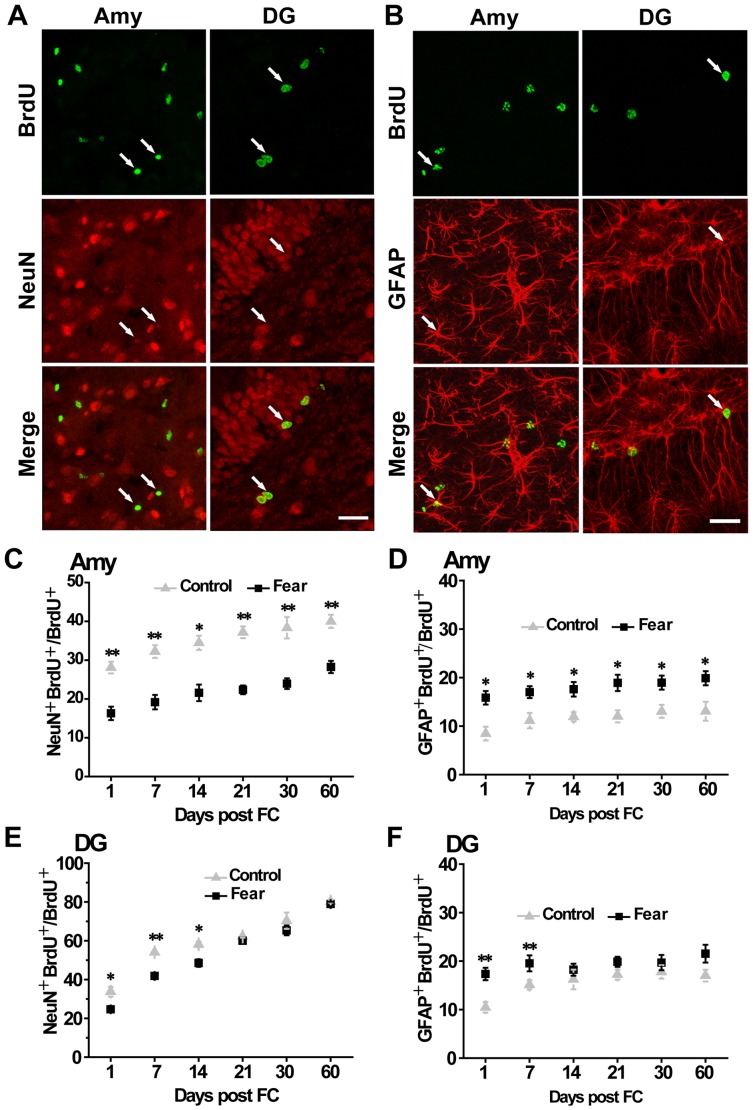
Auditory fear conditioning decreased neuronal maturation and increased gliogenesis in the amygdala and the dentate gyrus. Representative confocal scanning laser images of sections doubled-labeled with BrdU and NeuN (A) or GFAP (B) in the amygdala and the DG. Auditory fear conditioning reduced the number of adult-born cells that defferentiated into mature neurons (C) and increased gliogenesis (D) in the amygdala at 1 d (n = 3), 7 d (n = 3), 14 d (n = 3), 21 d (n = 3), 30 d (n = 3) and 60 d (n = 3) after the fear conditioning. Auditory fear conditioning transiently suppressed the maturation of adult-born cell into neurons (E) and increased gliogenesis (F) in the DG. * *P*<0.05, ** *P*<0.01. Scale bar = 30 µm.

To investigate whether auditory fear conditioning has any effect on the differentiation of adult-born cells, we performed double labeling for newly born cells with BrdU and the mature neuron maker NeuN or the astrocyte maker GFAP at different time points after auditory fear conditioning. Confocal microscopy confirmed the colocalization of BrdU and NeuN/GFAP in individual cells (Figure 2A, B). One day after fear conditioning, the ratio of BrdU-postive cells expressing NeuN was 16.28±1.73% in the amygdala in animals underwent fear conditioning compared to 28.1±1.52% in control animals (*P*<0.01) (Figure 2C **)**. In contrast, the proportion of BrdU-postive cells expressing GFAP was higher in fear rats than in controls (*P*<0.05) (Figure 2D). The same pattern of the BrdU-positive cells in fear rats was detected until 60 days after training, at which time point, 28.23±1.57% of BrdU-positive cells expressed NeuN in fear rats compared to 40±1.72% in controls (*P*<0.05); meanwhile, the proportion of BrdU-postive cells expressing GFAP was18.87±1.46% in fear rats compared with 13.05±1.95% in control animals (*P*<0.05). Similar results were obtained in the hippocampus in the first two weeks after fear conditioning, but the difference disappeared at later time points (Figure 2E, F). The results show that auditory fear conditioning decreased the differentiation of new-born cells into mature neurons but increased the differentiation of new-born cells into astrocytes in the amygdala.

### Fluoxetine Treatment Ameliorated the Impairment of Adult Neurogenesis Induced by Auditory Fear Conditioning and Suppressed the Maintenance of Auditory Fear Memory

**Figure 3:**
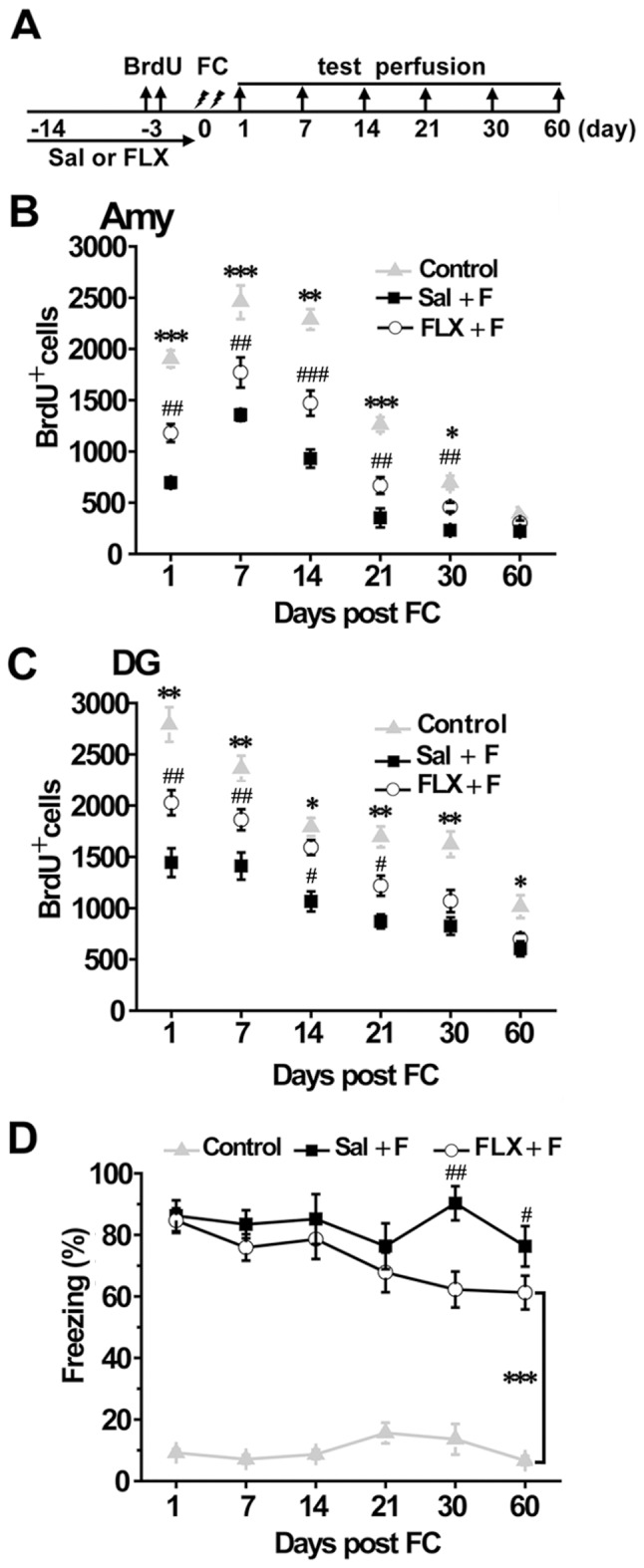
Fluoxetine treatment partially rescued the decrease of cell survival caused by auditory fear conditioning and suppressed the maintenance of long-term auditory fear memory. (A) Diagram of experimental procedures. (B) Fluoxetine treatment partially rescued the decrease of cell survival caused by auditory fear conditioning in the amygdala. The number of BrdU-positive cells was counted at 1 d (n = 3), 7 d (n = 3), 14 d (n = 3), 21 d (n = 3), 30 d (n = 3) and 60 d (n = 3) after training. There was a significant increase the number of BrdU-positive cells in the amygdala in FLX+F group compare to the Sal+F group at all time points, but the number of cells was still lower than in control group. Similar results were obtained for hippocampus (C). (D) Fluoxetine suppressed the maintenance of the long-term auditory fear memory. There was no difference between the two groups when auditory fear memory was measured at 1 d (n = 8), 7 d (n = 8) 14 d (n = 8) and 21 d (n = 8) after training. However, when measured at 30 d (n = 8) and 60 d (n = 8) after training, the freezing level in fluoxetine fear (FLX+F) rats was significantly lower than in the saline control fear (Sal+F) rats, but still higher than that in the controls. FC = fear conditioning; Sal = saline; FLX = fluoxetine. ^#^ or * *P*<0.05, ^##^ or **P<0.01, ^###^ or *** *P*<0.001. ^#^ FLX+F vs. Sal+F,* FLX+F vs. Control.

These above findings suggested that the new-born cells in the amygdala may contribute to persistent auditory fear memory. Chronic FLX treatment can enhance the survival of new-born cells and promote the maturation of hippocampal adult-born neurons [20]. Thus, we tried to promote amygdaliod neurogenesis by 2 w of FLX treatment before auditory fear conditioning (Figure 3A). Both the survival and maturation of new-born cells in the amygdala were improved in FLX treated rats. In addition, FLX treatment partly rescued the decrease in cell survival induced by auditory fear contidioning and completely reversed the alteration of cell differentiation in the amygdala. The ANOVA showed a significantly effect for group, and *post hoc t* tests showed a significant difference between Sal+F group and FLX+F group, as well as between FLX+F group and control group 1 d (F_(2,6) = _103.283, *P*<0.001), 7 d (F_(2,9)_ = 69.452, *P*<0.001), 14 d (F_(2,9)_ = 34.187, *P*<0.001), 21 d (F_(2,9)_ = 72.321, *P*<0.001) and 30 d (F_(2,9)_ = 20.238, *P*<0.001) after auditory fear conditioning. The number of BrdU-positive cells in FLX treated (FLX+F) rats was significantly increased at 1 d (Sal+F vs. FLX+F: 696±52.30 vs. 1180±87.18, *P*<0.01), 7 d (Sal+F vs. FLX+F: 1356±56.71 vs. 1771±147.12, *P*<0.01), 14 d (Sal+F vs. FLX+F: 930±89.73 vs. 1471±122.85, *P*<0.001), 21 d (Sal+F vs. FLX+F: 352±92.261 vs. 668±81.54, *P*<0.01) and 30 d (Sal+F vs. FLX+F: 231±44.46 vs. 457±42.35, *P*<0.01) time points but were still lower than naive controls (**Figure 3B**). Similar results were obtained in the hippocampus (**Figure 3C**).

**Figure 4:**
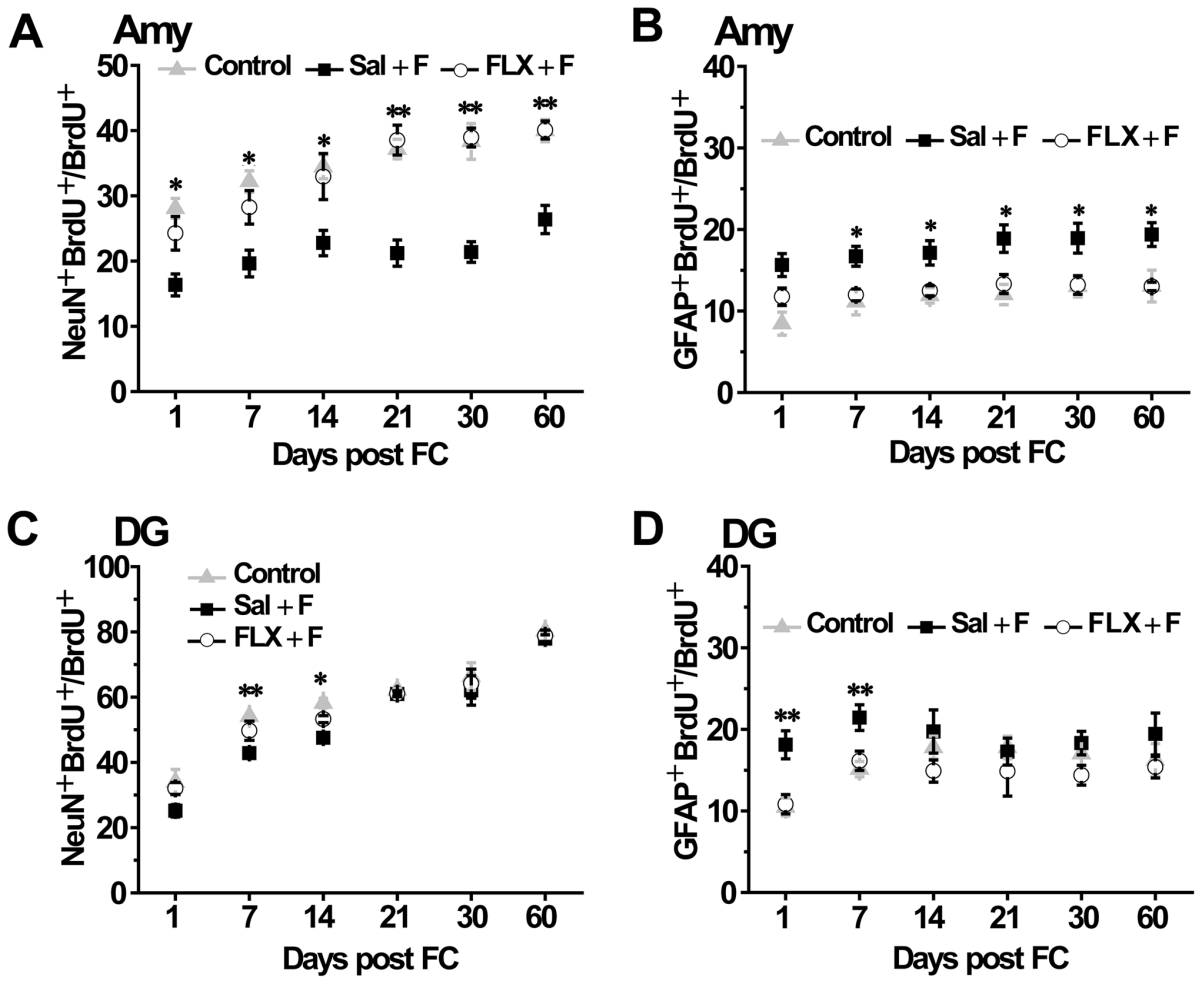
Fluoxetine treatment rescued the alterations in adult-born cell differentiation caused by auditory fear conditioning. Fluoxetine treatment completely rescued the decrease in the number of adult-born cells expressing NeuN (A) and the increase in the number of adult-born cells expressing GFAP (B) induced by auditory fear conditioning in the amygdala. The propotion of BrdU-positive cells co-expressing NeuN was significantly increased in fluoxetine pre-treated (FLX+F) rats compared to saline control fear (Sal+F) animals and reached to the level of the control group. Similarly, the propotion of BrdU-positive cells co-expressing GFAP was significantly decreased when measured at 1 d, 7 d, 14 d, 21 d, 30 d and 60 d after auditory fear conditioning. Similar results were obtained for the hippocampus (C, D). n = 3; * *P*<0.05, ** *P*<0.01.

The results of cell differentiation showed that the proportion of BrdU-postive cells expressing NeuN was obviouly different among three groups. The ANOVA showed a significantly effect for group, *post hoc t* tests showed a significant difference between Sal+F group and FLX+F group, but no difference between FLX+F group and control group 1 d (F_(2,6)_ = 10.467, *P*<0.05), 7 d (F_(2,6)_ = 11.923, *P*<0.01), 14 d (F_(2,6)_ = 7.035, *P*<0.05), 21 d (F_(2,6)_ = 24.059, *P*<0.01), 30 d (F_(2,6)_ = 27.654, *P*<0.01) and 60 d (F_(2,6)_ = 16.317, *P*<0.01) after auditory fear conditioning. Compared to saline control of the animals underwent fear conditioning, the proportion of BrdU-postive cells expressing NeuN was increased in FLX treated animals 1 d (Sal+F vs. FLX+F: 16.35±1.69% vs. 25.21±3.50, *P*<0.05), 7 d (Sal+F vs. FLX+F: 19.64±1.05% vs. 28.25±2.57, *P*<0.05), 14 d (Sal+F vs. FLX+F: 22.78±1.13% vs. 32.96±3.53, *P*<0.05), 21 d (Sal+F vs. FLX+F: 21.22±2.00% vs. 38.54±2.30, *P*<0.01), 30 d (Sal+F vs. FLX+F: 21.39±1.58% vs. 38.95±1.45, *P*<0.01) and 60 d (Sal+F vs. FLX+F: 26.38±2.16% vs. 40.09±1.39, *P*<0.01) after auditory fear conditioning (**Figure 4A**). The proportion of BrdU-postive cells expressing GFAP was also different among three groups. The ANOVA showed a significantly effect for group, *post hoc t* tests showed a significant difference between Sal+F group and FLX+F group, but no difference between FLX+F group and control group 7 d (F_(2,6)_ = 5.887, *P*<0.05), 14 d (F_(2,6)_ = 7.038, *P*<0.05), 21 d (F_(2,6)_ = 6.944, *P*<0.05), 30 d (F_(2,6)_ = 8.965, *P*<0.05) and 60 d (F_(2,6)_ = 6.494, *P*<0.05) after auditory fear conditioning. Compared with Sal+F group, the ratio of BrdU-postive cells expressing GFAP was significantly decreased in FLX+F group 7 d (Sal+F vs. FLX+F: 16.70±1.22% vs. 11.99±0.72, *P*<0.05), 14 d (Sal+F vs. FLX+F: 17.13±1.49% vs. 12.48±0.61, *P*<0.05), 21 d (Sal+F vs. FLX+F: 18.87±1.68% vs. 13.18±1.17, *P*<0.05), 30 d (Sal+F vs. FLX+F: 18.93±1.84% vs. 13.18±1.13, *P*<0.05) and 60 d (Sal+F vs. FLX+F: 19.36±1.46% vs. 13.01±0.51, *P*<0.05) after auditory fear conditioning (**Figure 4B**). The percent of BrdU-postive cells expressing both NeuN and GFAP in the hippocampus was similar to the corresponding percentages in control rats (**Figure 4C,D**).

As expected, we observed that FLX suppressed the maintenance of the long-term auditory fear memory. In the FLX and saline treated fear rats, auditory fear memory was similar at 1 d, 7 d, 14 d and 21 d after auditory fear conditioning (**Figure 3D**). However, auditory fear memory was significantly lower in FLX treated rats as compared with saline control at 30 d (*P*<0.01) and 60 d (*P*<0.05) after fear conditioning (**Figure 3D**). Thus, FLX partially rescued the impairment of adult neurogenesis in the amygdala and suppressed the maintenance of the long-term auditory fear memory.

## Discussion

The main findings of the present study were that the persistent auditory fear memory induced by fear conditioning led to decrease the survival and an alteration in the differentiation pattern of new-born cells in the amygdala. Chronic treatment with FLX before auditory fear conditioning partially rescued the impairment of the amygdaloid neurogenesis and suppressed long-lasting auditory fear memory in rats.

Fear conditioning is a widely used as a model of PTSD [4], [26]. Previous studies have shown that fear learning and traumatic stress reduced hippocampal neurogenesis [27], [28]. Hippocampal neurogenesis is involved in learning and maintainence of contextual fear memory [8], [9], [11], [29]. In studying of the neurogenesis in the amygdala, only a few concerned stress and amygdaloid adult-born cells were reported. Resently, Lieberwirth et al. found that the stressful social isolation reduced amygdaloid cell survival and neuronal differentiation in adult prairie voles [18]. Extending this finding, our data showed that auditory fear conditioning also significantly decreased cell survival and neuronal differentiation in the rat amygdala. Furthermore, our data showed that the alteration of neurogenesis was accompanied by enhanced gliogenesis because a higher proportion of astrocytes developed from the BrdU-positive cells. Similar alterations wese observed in the hippocampus, but the alteration was transient lasting about two weeks. The different effects of auditory fear conditioning on cell differentiation in the hippocampus and amygdala might indicate their different functions in auditory fear memory [30]. How the observed changes in the differentiation of newly generated cells would affect the functions of the amygdala remains largely unknown. Adult-born neurons in the hippocampus are involved in certain types of memory [8]–[10], new neurons in the olfactory bulb contribute to olfactory discrimination learning [31], and new neurons in the hypothalamus are involved in energy balance [32]. The amygdala is an important structure for the acquisition and storage of fear memory [4]. Thus, it is likely that amygdaloid adult-generated neurons are involved in memory processes. The decrease in the number of mature neurons and the increase in the number of astrocytes in the amygdala after auditory fear conditioning might relate to structural plasticity and lead to the persisitence of auditory fear memory. Conversely, enhancement of the adult neurogenesis by FLX treatment might lead the animal to better adapt the situation, leading to the suppression of the maintenance of the fear memory.

FLX treatment is known to increase adult neurogenesis in the rodent hippocampus [19]–[21]. Thus, we also tested whether FLX treatment before fear conditioning increased amygdaloid neurogenesis and whether amygdaloid neurogenesis was involved in the auditory fear memory. Our results showed that FLX treatment partially restored the decrease of neuronal survival and completely reserved the alteration of cell differentiation caused by auditory fear conditioning. Strikingly, FLX treatment suppressed auditory fear memory, although the effects were not observed untill 30–60 days after fear conditioning. The effects of FLX treatment could be attributed to the increased survival of amygdaloid cells and maturation of new-born neurons in the amygdala, perhaps leading to a better adaption to the situation. Hippocampal adult-born neurons functionally integrated into existing local circuits at 4–8 weeks of age [33]–[35]. Thirty days after fear conditioning, the new-born neurons in the amygdala were approximately 5-week old and might have been integrated into local circuits, producing more functional plasticity. Chronic FLX administration increases brain-derived neurotrophic factor (BDNF) expression in many brain areas [36], including the amygdala and hippocampus [22], and BDNF is a major mediator of the adult neurogenesis [37], [38]. Increased BDNF in the amygdala and hippocampus likely contributed to the partially rescue of the fear-conditioning-induced impairment of adult neurogenesis by FLX, but this will require further investigation. A recent report showed that the fear erasure requires FLX treatment combined with extinction training [22]. Our results are consistent with this report, but while FLX suppressed auditory fear memory, the freezing levels in FLX fear rats still higher than that of naïve rats. Adult neurogenesis could enable the dynamic remodeling of mature neuronal circuits by adding new neurons, contributing to structural and functional plasticity in the brain. FLX treatment ameliorated the impairment of amygdaloid neurogenesis aftere fear conditioning, and thus protected plasticity in the amygdala.

Our study also showed that the number of adult-born cells in the amygdala reached to peak at 7 days after fear conditioning, and two months after the fear conditioning, only about forty percent of BrdU-positive cells expressing NeuN in the amygdala in control rats, which suggested that adult-born cells in the amygdala have longer proliferation time periods and fewer cells attain a mature phenotype compared to those in the hippocampus. These data could contribute to a new understanding for the treatment of PTSD based on the distinct characteristics of the adult neurogenesis in the amygdala and hippocampus.

In the study, we used BrdU to investigate the relationship between amygdaloid neurogenesis and auditory fear memory. BrdU can incorporate DNA of dividing cells during the S-phase of the cell cycle, so not all BrdU-labeled cells are neural cells, which is the limitation of BrdU, through BrdU-labeling is the most used method for studying adult neurogenesis.

## Conclusions

The present study used auditory fear conditioning to elucidate the relationship between amygdaloid neurogenesis and auditory fear memory. The results demonstrate that auditory fear conditioning induced persistent auditory fear memory, which was associated with suppression of the survival and maturation of new-born cells in the amygdala. FLX treatment partially reversed the impairments in amygdaloid neurogenesis, leading to the suppression of long-term fear memory. Overall, our data suggest that adult neurogenesis in the amygdala may be contribute to auditory fear memory.
